# Prevalence and Follow-Up of Occult HCV Infection in an Italian Population Free of Clinically Detectable Infectious Liver Disease

**DOI:** 10.1371/journal.pone.0043541

**Published:** 2012-08-22

**Authors:** Laura De Marco, Paola Manzini, Morena Trevisan, Anna Gillio-Tos, Franca Danielle, Cinzia Balloco, Alessandra Pizzi, Eleonora De Filippo, Sergio D’Antico, Beatrice Violante, Adriano Valfrè, Franco Curti, Franco Merletti, Lorenzo Richiardi

**Affiliations:** 1 Unit of Cancer Epidemiology, C.E.R.M.S., University of Turin, Turin, Italy; 2 Blood Bank, A.O.U. S. Giovanni Battista, Turin, Italy; 3 Center for Oncologic Prevention, University of Turin, Turin, Italy; Centers for Disease Control and Prevention, United States of America

## Abstract

**Background:**

Occult hepatitis C virus infection (OCI) is a recently described phenomenon characterized by undetectable levels of HCV-RNA in serum/plasma by current laboratory assays, with identifiable levels in peripheral blood mononuclear cells (PBMCs) and/or liver tissue by molecular tests with enhanced sensitivity. Previous results from our group showed an OCI prevalence of 3.3% in a population unselected for hepatic disease. The present study aimed to evaluate OCI prevalence in a larger cohort of infectious liver disease-free (ILDF) subjects. Clinical follow-up of OCI subjects was performed to investigate the natural history of the infection.

**Methods and Findings:**

439 subjects referred to a Turin Blood Bank for phlebotomy therapy were recruited. They included 314 ILDF subjects, 40 HCV-positive subjects and 85 HBV-positive subjects, of whom 7 were active HBV carriers. Six subjects (4/314 ILDF subjects [1.27%] and 2/7 active HBV carriers [28%]) were positive for HCV-RNA in PBMCs, but negative for serological and virological markers of HCV, indicating OCI. HCV genotypes were determined in the PBMCs of 3/6 OCI subjects two had type 1b; the other had type 2a/2c. OCI subjects were followed up for at least 2 years. After 12 months only one OCI persisted, showing a low HCV viral load (3.73×10^1^ UI/ml). By the end of follow-up all OCI subjects were negative for HCV. No seroconversion, alteration of liver enzyme levels, or reduction of liver synthesis occurred during follow-up.

**Conclusions:**

This study demonstrated the existence of OCI in ILDF subjects, and suggested a high OCI prevalence among active HBV carriers. Follow-up suggested that OCI could be transient, with a trend toward the decrease of HCV viral load to levels undetectable by conventional methods after 12–18 months. Confirmation studies with a longer follow-up period are needed for identification of the OCI clearance or recurrence rates, and to characterize the viruses involved.

## Introduction

In the past years, a new form of hepatitis C virus (HCV) infection has been identified and defined as “occult” HCV infection (OCI) [1]–[3]. OCI is characterized by: i. detection of HCV-RNA in liver tissue alone, or in liver tissue and/or peripheral blood mononuclear cells (PBMCs); ii. consistently undetectable HCV-RNA in serum [4]–[5].

OCI has two possible sets of clinical features: negativity for both serum anti-HCV antibodies (anti-HCV) and HCV-RNA with abnormal liver function tests, or positivity for serum anti-HCV and no detectable HCV-RNA with normal liver enzyme levels years after spontaneous, or therapy-induced resolution of HCV infection [2], [4]–[7]. We have also reported on OCI in subjects without evidence of hepatic disease [8]. The reason why HCV-RNA is not detectable in the serum of OCI patients is unknown. One hypothesis is that the number of circulating viral particles in OCI patients is too low to be detected by conventional molecular techniques [9]. To overcome this limitation, the sensitivity of detection methods has been improved by introducing ultracentrifugation of plasma, or mitogen *in vitro* stimulation of PBMCs [2], [10]. However, despite these efforts, HCV-RNA was not detected in these patients.

HCV has been shown to infect, and replicate in, both liver tissue and PBMCs of OCI patients, as indicated by the antigenomic HCV-RNA strand found in these patients [11]. In spite of its relatively recent identification, OCI has been investigated in different geographical regions [12], with emerging implications in different clinical scenarios. Barril and colleagues found an OCI prevalence of 45% in 109 haemodialysis patients [13], and the same group of investigators claimed there was a potential transmission risk of OCI [14], as they found a high OCI prevalence among relatives of OCI patients, comparable to that found among family members of patients with chronic HCV infection. Conversely, other authors did not find OCI in immune-suppressed subjects [15]–[16], in 28 onco-haematological [17] patients, or in 26 kidney-transplant patients [18].

Discordant OCI results in other HCV-related clinical manifestations, such as mixed cryoglobulinemia [19]–[20], autoimmune disorders [4] and non-Hodgkin lymphoma [21], have been published, and suggest that more studies in this field are needed. To our knowledge, no data have been published on OCI in patients with hepatitis B virus (HBV) infection.

The OCI prevalence in the general population is presently unknown. We previously reported on the occurrence of OCI in a population unselected for hepatic disease: in a sample of 276 apparently healthy Italian subjects who were negative for serological markers of HCV and viraemia, some 3% harboured HCV-RNA in their PBMCs [8]. In order to further investigate this unexpected finding, the present study aimed to evaluate the OCI prevalence in a broader series of infectious liver disease-free (ILDF) subjects, through testing of serial samples and long-term clinical follow-up. We also investigated HCV genotypes, HCV-RNA replication potential, and clearance or persistence of the virus in PBMCs from serial blood samples.

## Results

Characteristics of the 439 subjects included in the study (336 men and 103 women, ratio 3∶1, mean age: 67 years) are shown in Table 1. The study population was selected for diseases treated by phlebotomy therapy (hereditary hemochromatosis, iron overload, porphyria cutanea tarda, secondary erythrocytosis, polycythemia vera and arteriopathy), and was composed of subjects with a high mean age and a wide age range (26–88 years); the subgroup of subjects treated for iron overload were the youngest (mean age 61.8 years). We found altered alanine aminotransferase (>50 IU/L for men and >35 IU/L for women) and aspartate aminotransferase (>40 IU/L for men and >25 IU/L for women) levels in 57 men and 35 women respectively, with a higher frequency in the HCV-positive control group than in the ILDF group (p<0.0001; data not shown).

**Table 1:** Characteristics of the study subjects.

	INFECTIVE LIVER DISEASE-FREE GROUP (ILDF) (N = 314)	HCV-POSITIVE CONTROL GROUP (N = 40)	HBV-POSITIVE GROUP (N = 85)
**DIAGNOSIS**			
Hereditary hemochromatosis	38	7	9
Iron overload	28	16	8
Porphyria cutanea tarda	1	3	1
Secondary erythrocytosis	103	5	30
Polycythemia vera	140	9	36
Arteriopathy	4	0	1
**AGE**			
Range (mean) years	26–82 (65.25)	41–85 (64.4)	36–88 (66.7)
26–40 years	16	0	2
41–55 years	55	14	7
56–70 years	132	10	34
71–85 years	107	16	40
>86 years	4	0	2
**GENDER**			
Male/Female	235/79	34/6	67/18

None of the 439 subjects tested positive for markers of human immunodeficiency virus (HIV1–2) infection (HIV1–2 antibodies in serum and HIV-RNA in plasma).

Three groups of subjects were generated based on viral infection and serological pattern: ILDF, HCV-positive control group and HBV-positive group.

**Figure 1:**
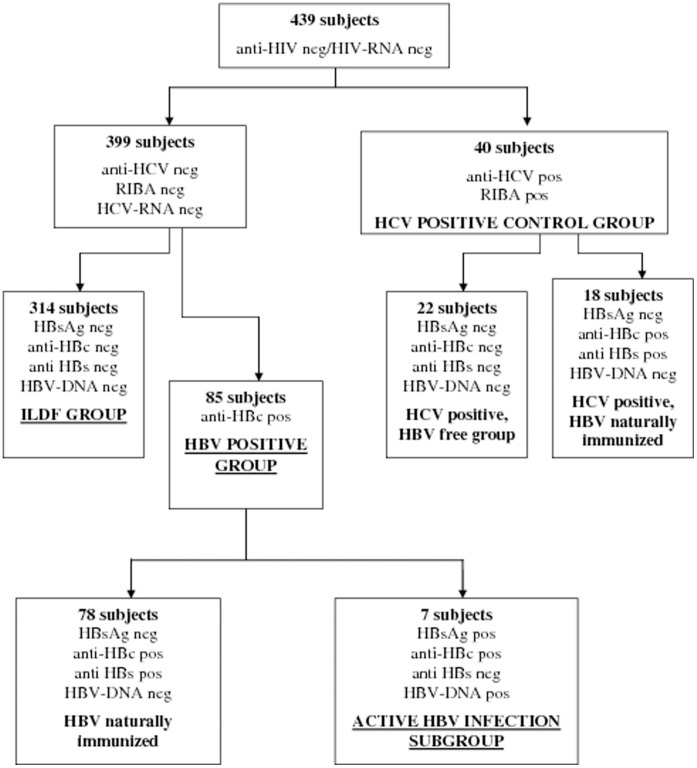
Flow chart of distribution of subjects enrolled in the study based on presence of serological markers of human immunodeficiency virus (HIV), hepatitis C virus (HCV) and hepatitis B virus (HBV). Subjects positive for HBsAg and/or HBV-DNA were considered carriers of HBV. Subjects positive for anti-HCV and HCV-RNA or anti-HCV positive confirmed by the RIBA assay even though negative for HCV-RNA were considered HCV infection. Subjects HBsAg negative, anti-HBc positive with or without anti-HBs and HBV-DNA negative with normal alanine aminotransferase levels were considered previously HBV infected and naturally immunized. Abbreviations: anti-HIV, HIV antibodies; anti-HCV, antibodies against HCV; HBsAg, hepatitis B surface antigen; anti-HBc, hepatitis B core antibodies; pos, positive; neg, negative.

The ILDF group included 314 subjects negative for markers of both HBV and HCV infection in serum (hepatitis B surface antigen, HBsAg and anti-HCV) and plasma (HBV-DNA and HCV-RNA), and for markers of previous HBV contact (antibodies against hepatitis B surface antigen, anti-HBs; antibodies against hepatitis B core antigen, anti-HBc) (Figure 1).

The HBV-positive group included 85 subjects who were negative for previous or active HCV infection (anti-HCV/HCV-RNA). Seventy-eight of the subjects in the HBV-positive group showed a resolved HBV infection (negative for HBsAg/HBV-DNA), while seven still had an active HBV infection (positive for HBsAg/HBV-DNA) (Figure 1). These seven subjects (all men; mean age 68.7 years) were also placed into an “active HBV infection subgroup”.

Among the seven subjects in the active HBV infection subgroup, five were HBsAg-positive in serum and HBV-DNA-positive in plasma, and one was HBsAg-positive but HBV-DNA-negative because he was on interferon treatment. The remaining subject in the active HBV infection subgroup was positive for HBV-DNA in plasma but negative for HBsAg in serum (the serological pattern of occult HBV infection).

The HCV-positive control group included 40 subjects (34 men and 6 women; mean age 64.4 years) who were anti-HCV positive by a RIBA assay, 38 of whom were already known to be infected. (Figure 1). Eighteen of them also showed markers of a resolved HBV infection (positive for anti-HBc; negative for HBsAg/HBV-DNA).

No subjects in the active HBV infection subgroup or the HCV-positive control group were taking interferon treatment during the study period.

HCV-RNA was detectable in the plasma of 34 out of the 40 subjects in the HCV-positive control group. None of the subjects in the ILDF group or the HBV-positive control group were HCV-RNA-positive in plasma. The absence of HCV-RNA in these subjects was confirmed after ultracentrifugation of plasma.

Of the 34 subjects with HCV-RNA in plasma, genotyping of the isolated HCV-RNA showed that 18 (53%) had genotype 1b, three (9%) had genotype 1a, three (9%) had genotype 2, six (18%) had genotype 2a/2c, two (6%) had genotype 4d, one (3%) had genotype 3a and one (3%) had genotype 1a/1b (data not shown). In addition, 30 out of the 34 subjects who were HCV-RNA-positive in plasma were also HCV-RNA-positive in PBMCs (Table 2), and the genotypes found therein were the same as those found in plasma, save one subject who had HCV 1a in plasma and 1b in PBMCs.

**Table 2:** HCV-RNA detection in plasma and PBMCs.

PLASMA HCV-RNA	HCV-RNA PBMCs POSITIVE	HCV-RNA PBMCs NEGATIVE		TOTAL	
**Infective liver disease-free group**					
Positive	0	0		0	
Negative	4	310		314	
**Total**	**4**	**310**		**314**	
**HCV-positive control group**					
Positive	30		4		34
Negative	0		6		6
**Total**	**30**		**10**		**40**
**HBV-positive group**					
Positive	0		0		0
Negative	0		85		85
**Total**	**0**		**85**		**85**
**active HBV infection subgroup**					
Positive	0		0		0
Negative	2		5		7
**Total**	**2**		**5**		**7**

Abbreviations: HCV, hepatitis C virus; PBMC, peripheral blood mononuclear cells; HBV, hepatitis B virus.

A total of six subjects who were negative for all markers of HCV infection in serum and plasma, were positive for HCV-RNA in PBMCs by gel electrophoresis and by real-time PCR (Table 2), thereby revealing OCI: four (1.27%) of the 314 subjects in the ILDF group and two (28%) of the seven subjects in the active HBV infection subgroup.

HCV genotypes in PBMCs were determined for three of the six OCI subjects, resulting in HCV type 1b in two cases, and 2a/2c in another (Table 3). Three samples failed to be genotyped due to weak detection of the PCR fragment.

**Table 3:** Serological and virological characteristics of subjects with occult hepatitis C virus (HCV) infection.

N	SEX	AGE	DISEASE	HBV status	AST ALT*	GGT ALP**	Liver functional tests***	HCV antibodies	HCV-RNA plasma	HCV-RNA PBMCs	HCV genotype	VIRAL LOAD PBMCs§ (IU/ml)
**1**	M	71	SE	neg	−/−	−/−	normal	−	−	+	n.d.	1,55E+02
**2**	M	57	SE	neg	−/−	−/−	normal	−	−	+	n.d.	6,47E+01
**3**	M	69	SE	POS	−/−	−/−	normal	−	−	+	n.d.	1,40E+02
**4**	F	28	PV	neg	+/+	−/−	normal	−	−	+	2a/2c	1,71E+04
**5**	F	81	SE	neg	−/−	−/−	normal	−	−	+	1b	3,24E+02
**6**	M	73	SE	POS	−/−	−/−	normal	−	−	+	1b	2,39E+05

* AST and ALT values at recruitment; −/−: normal values of AST: below 40 IU/L and 32 IU/L for men and women respectively, and ALT: below 50 IU/L and 35 IU/L for men and women respectively. +/+ altered levels of AST and ALT.

** GGT and ALP values; −/−: normal values of GTT: below 50 IU/L and 35 IU/L for men and women respectively, and ALP: below 128 IU/L and 141 IU/L for men and women respectively.

*** Liver functional tests: total plasma protein, albumin, cholesterol, total and fractionated bilirubin, prothrombin time, partial thromboplastin time, and international normalised ratio.

§ PBMCs viral load on baseline sample.

Abbreviations: SE, secondary erythrocytosis; PV, polycythemia vera; HBV, hepatitis B virus; AST, aspartate aminotransferase; ALT, alanine aminotransferase; GGT, gamma glutamyl transpeptidase; ALP, alkaline phosphatase, n.d., not determined because the amplification resulted in a very weak fragment and sequence analysis did not succeed; PBMCs, peripheral blood mononuclear cells.

At the end of recruitment, all six OCI subjects were followed up until March 2011 (Table 4). Liver function tests were not altered in any of the OCI subjects. Liver enzyme levels remained within normal ranges for five OCI subjects throughout the study period. Only one of the six OCI subjects showed slightly abnormal alanine aminotransferase levels (54 IU/L) at recruitment, which subsequently decreased to normal levels during follow-up. Negativity for anti-HCV in serum and HCV-RNA in plasma persisted during follow-up for all subjects, and was further confirmed after ultracentrifugation of plasma. When PBMCs from follow-up specimens were tested by reverse-transcription PCR assay, negative results were found at gel electrophoresis evaluation, which has known limits in the detection of low density (<25 ng) amplicons. All 42 follow-up samples from the six OCI subjects were then retested by real-time PCR, and three turned out to be positive (Table 4).

**Table 4:** Timing of blood sampling and HCV-RNA results on PBMCs during follow-up of OCI subjects.

	2008			2009						2010					2011		
N	April	May	Jul	Feb	Mar	Apr	May	June	Jul	Aug	Sep	Oct	Nov	Dec	Jan	Feb	Mar
1	1.55×10^2^				*2.51×10^2^*		N		N	N		N		N	N		N
2		6.47×10^1^		*N*		*N*					*N*	*N*		*N*	*N*		*N*
3		1.40×10^2^			*2.05×10^1^*			*3.73×10^1^*			*N*	*N*	*N*	*N*	*N*		
4			1.71×10^3^				*N*				*N*	*N*		*N*			*N*
5					3.24×10^2^						*N*	*N*	*N*		*N*	*N*	*N*
6						2.39×10^3^						*N*	*N*		*N*		*N*

NOTE: Normal characters indicate baseline samples. Italics indicates follow-up samples.

Abbreviations: N, negative result.

HCV viral load was evaluated both in the plasma and PBMCs of the HCV-positive control group, and in the PBMCs of all samples collected from OCI subjects at recruitment and at follow-up, and the results reported in IU/ml. At recruitment, in the 34 subjects positive for HCV-RNA in plasma, the HCV viral load therein ranged from 4.60×10^3^ IU/ml to 7.73×10^3^ IU/ml, with a median of 1.73×10^7^ IU/ml. The amounts at the 25th and 75th percentiles were 1.51×10^4^ and 5.36×10^6^ IU/ml, respectively. In buffy-coats the viral load ranged from 1.11×10^1^ IU/ml to 2.49×10^6^ IU/ml, with a median of 2.25×10^5^ IU/ml. The values at the 25th and 75th percentiles were 1.19×10^4^ IU/ml and 5.30×10^5^ IU/ml, respectively (data not shown).

HCV viral loads for the six OCI subjects are shown in Table 3. The buffy-coat samples that were positive at baseline showed low viral loads, with a median of 2.40×10^2^ IU/ml (range from 6.47×10^1^ IU/ml to 2.39×10^3^ IU/ml). Ten months after recruitment only two subjects were still positive for HCV-RNA in PBMCs by quantitative PCR, with HCV-RNA levels of 2.51×10^2 ^IU/ml and 2.05×10^1^ respectively (subjects 1 and 3, Table 4). Twelve months after recruitment only one subject (subject 3) was still positive (HCV viral load of 3.73×10^1 ^IU/ml), but became negative by 25 months of follow-up (Table 4).

To investigate the potential transmission risk of OCI, we performed mitogen *in vitro* stimulation of about 5×10^6^ PBMCs from the six OCI subjects with Interleukin-2 and phytohaemoagglutinin for 7 days, and assessed the release of HCV virions in the supernatant. PBMCs from one subject in the HCV-positive control group, with an HCV viral load of 3.70×10^5^ IU/ml, and from two subjects in the ILDF group, were analogously stimulated and used as positive and negative controls, respectively. The positive control sample released the virus into the supernatant (1.99×10^1^ IU/ml). Only one OCI sample (subject 5, PBMCs HCV viral load 3.24×10^2^ IU/ml) showed a quantifiable amount of HCV genome in the culture medium by real-time PCR, corresponding to 2.2×10^1^ IU/ml. The negative controls and all other recruitment and follow-up samples from OCI subjects failed to show the virus in the supernatant.

## Discussion

Little is known about the prevalence of OCI, its natural history, potential transmission risk and impact in the general population. Detection of HCV-RNA in PBMCs in the absence of serological markers of HCV has been almost exclusively reported for patients with liver disease or immunosuppressed patients [1], [13], [22]. However, we recently found an OCI prevalence of 3.3% in a population of 276 subjects unselected for hepatic disease [8]. Here, in a broader series of subjects submitted to phlebotomy therapy for different disorders unrelated to infectious liver disease, we again confirmed the occurrence of OCI, with a prevalence of 1.27%. Furthermore we observed a high OCI prevalence (28%) (p = 0.004) in the active HBV infection subgroup. Markers of previous HBV infection were detected in several of our study subjects (103 total: 85 in the HBV-positive group, and 18 in the HCV-positive control group) (Figure 1). Among them, 20 (19.4%) had an HCV infection (18 overt and 2 OCI). When we considered subjects with HCV infection (46 total: 40 overt and 6 OCI) we found 20 subjects (43.5%) that had been infected with HBV.

The association observed in the present study between HCV and HBV infection is not a completely new finding. Occult HBV infection has also been described, with different prevalences in different studies, in HCV carriers [23], and chronic co-infection with the two viruses has been proposed as a factor that increases the risk of neoplastic progression [24].

If we consider the subjects with either resolved or active HBV infection, the prevalence of OCI was 1.94% (2/103), comparable with that found in the ILDF group. In this context HBV contact does not seem to be a risk factor for OCI. Conversely, the high prevalence of OCI (2/7) that we found in the active HBV infection subgroup is very intriguing and needs to be thoroughly investigated. Nevertheless, even though we found a statistically significant difference (p = 0.0008) within this small subgroup, any statement that active HBV infection is strongly associated with OCI must be considered with caution. One possible explanation for the high prevalence we observed is that active HBV infection could have a modulating effect on the immune system, facilitating the persistence of HCV infection in PBMCs. The fact that among all OCI subjects, the two with HBV showed the longest persistent infection (subject 3), and the highest viral load in PBMCs (subject 6), respectively, could support this hypothesis.

The OCI prevalence found in the ILDF group was lower than that found in our previous study (1.27% vs 3.3%). In addition, the latter population was not screened for markers of HBV infection, and because there is an intermediate prevalence of HBV infection in Italy that varies widely from region to region, we are not able to estimate how many subjects in our previous study were HBV carriers. Though the number of subjects was limited, considering the high OCI prevalence detected in the active HBV infection subgroup of the present study (28%), the higher prevalence found in our previous study could be explained by the presence of some HBV carriers.

No single HCV genotype was predominant in the present study; instead different genotypes were present in both the HCV-positive control group (1a, 1b, 2a, 3a, 4d) and in OCI subjects (1b, 2a/2c). Similar results have been reported in other case-series of subjects with spontaneous, or treatment-induced resolution of viral infections [2]. A different HCV genotype in PBMCs and plasma was found in only one subject from the HCV-positive control group. An analogous feature has been described by several authors [25]–[27], who reported that a proportion of HCV-infected subjects harboured genotypes in their PBMCs that could not be detected in their plasma. An explanation of these differences in the distribution of genotypes may be related to the presence of viral variants due to mutations that confer distinct cellular tropism [26], [27]. Studies of the hypervariable E2 region of HCV showed that specific variants are frequent, particularly in B lymphocytes and monocytes [25], [27].

A strength of our study was the follow-up of OCI subjects to partially investigate the natural history of the infection. The six subjects with OCI showed a very low HCV viral load in PBMCs, detected through highly sensitive methods, which in some cases did not even allow us to trace the genotype. Only two of these OCI subjects (subjects 1 and 3) had detectable HCV infection in PBMCs at 11 and 13 months of follow-up, respectively, demonstrating a persistent infection. There were two OCI subjects with HBV infection: one showed the longest persistent infection (subject 3), and the other had the highest viral load in PBMCs (subject 6) of all the OCI subjects, suggesting a potential interference between the two infections.

In all the other subjects (subjects 2, 4, 5 and 6) we failed to demonstrate OCI persistence, since none were HCV-RNA-positive in PBMCs at 9, 11, 18 and 25 months of follow-up, respectively, after initial detection. Moreover, after mitogen in vitro stimulation of PBMCs, no HCV was detectable, which points more to the actual clearance of the virus than to a fluctuating replication.

This is in accordance with recently published data describing 16 OCI subjects, detected after successful response to antiviral treatment, who cleared the virus from PBMCs at 6 to 12 months of follow-up [28]. Furthermore the apparent clearance of viremia in PBMCs in our subjects was not drug-influenced because none of our OCI subjects was undergoing interferon or ribavirin therapy during the study. At variance with our results is a study conducted by Castillo and colleagues, which reported on the follow-up of 37 subjects with a diagnosis of OCI who presented abnormal liver function of unknown aetiology for a minimum of 12 months [29]. The authors described the persistence of very low levels of HCV-RNA intermittently detected in PBMCs and in the serum of these patients during a mean follow-up of 55.7 months. In our subjects HCV viremia was never detected and HCV-RNA in PBMCs was never detected in follow-up samples after its disappearance. This pattern is more consistent with a possible clearance of the infection than with a persistent infection with replication flares, although the latter cannot be excluded. A longer follow-up is needed for confirmation.

HCV-RNA monitoring in serial PBMCs and serum improved the identification of OCI, and this approach has been suggested as a useful method for the diagnosis of OCI when a liver biopsy is not available [29].

The novelty of our findings lies in the transient detection of OCI for a mean follow-up time of 23 to 35 months in a population naïve for hepatic viral infections and without abnormal liver function.

Evolution of molecular assays and ultracentrifugation methods to improve the monitoring of HCV infection presently ensures detection of low levels of HCV-RNA. However, the relevance of HCV-RNA detection in PBMCs alone is poorly understood, although the proven ability of HCV to replicate in these cells [11], [30] raises questions about the potential transmission risk to other cell types (i.e., liver), or to other individuals through blood transfusions or haemodialysis. Indeed, the possible transmission of infection between OCI carriers and people undergoing haemodialysis has been described [13]. Among all enrolment and follow-up PBMCs in the present study, in only one sample was it possible to identify HCV-RNA in the supernatant after mitogen in vitro stimulation, showing the ability of the virus to replicate. Whether the HCV-RNA detected in supernatant represents competent viruses released from PBMCs is only presumable, but this could represent a potential transmission risk to different compartments, such as liver cells. Nevertheless no clinical evidence of liver involvement was seen in our subjects during follow-up. Moreover, for the aforementioned subject, no subsequent PBMC sample allowed for viral detection, suggesting that replication capability was not sufficient to maintain a persistent infection. The inability of the other samples to show detectable viraemia in the supernatant could be due to the very low number of virions originally infecting the PBMCs in our subjects, or to a low virus replication efficiency due to peculiar characteristics of the viruses. Further studies that perform full-length sequences of these viruses are needed to help show whether genomic mutations could cause this kind of impaired replication.

In conclusion, we observed an OCI prevalence of 1.27% in 314 ILDF subjects with no signs of active or previous viral hepatic infection, and an unexpectedly high OCI prevalence in HBV carriers. OCI seems unable to affect liver cells, or to have an impact on liver function, nor does it seem to produce an antibody immune response. It seems to be a transient infection that can potentially be cleared within 18 months of its detection. In this scenario, it seems unlikely that OCI could be easily transmitted between humans, although confirmation and family studies with a longer follow-up are needed to monitor viral clearance and potential transmission risk.

We are planning to continue follow-up of HCV-negative subjects and OCI subjects at 12-month intervals to investigate whether periodical recurrences of HCV infection with low viral loads occur in PBMCs, and if cumulative prevalence in the general population is higher than we detected.

## Materials and Methods

### Study Series

Between April 2008 and September 2009, we recruited a total of 439 subjects referred to the Blood Bank of the S. Giovanni Battista Hospital in Turin, Italy for phlebotomy therapy as treatment for different diseases (Table 1). A peripheral blood sample for PBMC separation (about 14 mL) was collected at enrolment into two tubes containing EDTA. Subjects were tested for liver enzyme levels, the presence of markers of HBV infection (HBsAg, anti-HBs, and anti-HBc), anti-HCV and HIV1–2 antibodies in serum samples, and for HBV-DNA, HCV-RNA and HIV-RNA in plasma. Subjects with OCI were followed-up for 23 to 35 months, during which they were prospectively tested at least six times for liver enzyme levels, anti-HCV in serum, and HCV-RNA in plasma and PBMCs.

The first follow-up sample was used to perform a complete hepatic synthesis, including dosage of total plasma protein, albumin, cholesterol, total and fractionated bilirubin, prothrombin time, partial thromboplastin time and international normalized ratio. All blood parameters were evaluated by standardized methods. Signed consent forms were obtained at the time of recruitment, and the study was approved by the local ethics committee (CEI, Comitato Etico Interaziendale S. Giovanni Battista Hospital and CTO-Maria Adelaide Hospital, Turin, Italy).

Plasma and buffy-coats were separated from 10 mL of whole blood and stored immediately at −80°C. PBMCs were isolated from the remaining 4 mL of whole blood using the Ficoll-Hypaque density gradient (Lonza, Walkersville, Maryland, USA). The PBMC pellet was then washed twice in phosphate-buffered saline (pH 7.4), and resuspended in RPMI 1640 medium supplemented with penicillin, streptomycin, glutamine and 50% heat-inactivated Foetal Calf Serum (FCS); an equal volume of a solution including RPMI 1640 (30%), dimethyl sulfoxide (DMSO, 20%), and FCS (50%) was added for alive freezing of PBMCs at −80°C.

### Detection of Markers of Hepatitis Infection in Serum Samples

HBsAg, anti-HBs, anti-HBc and anti-HCV testing in serum was performed by chemiluminescent microparticle immunoassay (CMIA) (Architect system, ABBOTT Diagnostic Division, Abbot Park, Illinois, USA); HIV1–2 antibodies were detected using CMIA for the simultaneous qualitative detection of HIV-p24 antigen and HIV1–2 antibodies (Architect system).

### Genomic Viral Detection in Plasma

Presence of HBV-DNA, HCV-RNA and HIV-RNA in plasma was detected using a multiplex transcription-mediated amplification technology (Procleix Ultrio on Tigris, Chiron, Emeryville, California, USA) with a 95% detection limit of 8–10 IU/ml for HBV-DNA, 3.01–3.17 IU/ml for HCV-RNA and 20–30 IU/ml for HIV-RNA.

### HCV-RNA Extraction and Detection in PBMCs

Total RNA was isolated from 150 µL of buffy-coat using the SV Total RNA Isolation System (Promega, Madison, Wisconsin, USA) and EXTRAgene (Amplimedical, Turin, Italy), respectively, according to the manufacturer’s instructions. In 150 µL of buffy-coat a cell number ranging from 1×10^6^ to 1×10^7^ was found. Buffy-coats were washed three times using SV RNA Red Blood Cell Lysis Solution (Promega) in order to avoid carry-over of inhibitors. Adequacy of extracted RNA was checked through reverse transcription of 5 µL to cDNA (Reverse Transcription System, Promega) and amplification of a 171 bp fragment of the beta-actin gene as previously reported [8].

Detection of HCV-RNA was performed by a commercial reverse-transcription nested-PCR system (Alfa Wasserman, Milan, Italy) using the primers targeting the 5′UTR region of the HCV genome included in the kit as previously described [8]. The presence of an 187 bp amplification fragment indicated HCV-RNA positivity in the sample. Synthetic HCV-RNA corresponding to the 5′UTR region, HCV-negative human serum, and RNA-free samples were included as positive and negative controls, respectively, in each PCR run. The sensitivity of the test was up to 50 genome equivalents (6 IU/ml). Repeat testing was performed on all positive samples for confirmation in different PCR amplification runs to exclude the possibility of contamination.

### HCV-RNA Detection in Ultracentrifuged Plasma Samples

Plasma samples from HCV-RNA-negative subjects were subjected to ultracentrifugation to allow detection of low amounts of viral particles. Two mL of stored plasma were ultracentrifuged for 3 hours at 100,000 g at 4°C, and RNA extracted from the resulting pellet was used for HCV-RNA detection.

### HCV Viral Load

Ten µL of purified RNA were used to evaluate HCV viral load by amplification in real-time PCR (artus®HCV RG RT-PCR kit, Qiagen, Hilden, Germany) on the iCycler iQ™ Real-Time PCR Detection System (Bio-Rad, Hercules, California, USA) as follows: one cycle of reverse transcription at 50°C for 30 minutes; 13 minutes at 95°C followed by 50 cycles of denaturation for 30 seconds at 95°C; annealing for 1 minute at 55°C; and extension for 30 seconds at 72°C. Each amplification run included negative and positive controls for HCV-RNA to monitor the performance of the methods, as well as a curve of calibrators (10^4^, 10^3^, 10^2^, and 10^1^ IU/µL) purchased with the kit. The sensitivity of the test was 34 IU/ml. A further point of 1 IU/µL (about 3.4 IU/ml) was created by 10-fold dilution of the 101 calibrator to improve reliable quantification of low viral loads.

The threshold cycle (Ct) was calculated by a software for data analysis with an automatic baseline setting (BioRad). An external calibration curve was generated automatically by plotting the Ct values against the logarithm of the IU numbers of the serial calibrators carrying the HCV genome. For quantification, the absolute IU number of unknown samples was calculated by plotting the Ct values against the logarithm of the standard curve. The sensitivity of the method was 10^−1^ IU. HCV viral load was expressed in IU/ml according to the following formula: Result (IU/mL) = [Result (IU/µL)×Elution Volume (µL)]/Sample Volume (mL).

### HCV Genotyping

HCV genotyping was performed in plasma samples using the VERSANT® HCV Genotype 2.0 kit (Siemens Medical Solution Diagnostic, Tarrytown, New York, USA) according to the manufacturer’s instructions. This system is a line-probe assay based on reverse hybridization. It identifies HCV genotypes 1 to 6 and subtypes “a” and “b” of genotype 1; it has a sensitivity of 96%, and a concordance with sequence-based phylogenetic analysis of 100%.

HCV genotyping for buffy-coat-derived PCR amplicons was performed by direct sequencing, after excision from the gel of the 187 bp 5′UTR fragment and purification with the “PCR Clean-up Gel Extraction Kit” (Macherey-Nagel, Dueren, Germany). Direct sequencing was carried out using the Big Dye Terminator Cycle Sequencing Ready Reaction Kit (Applera, Monza, Italy), and sequence analysis was performed with the ABI 310 automated capillary system (Applera), following the manufacturer’s instructions. Sequences were analysed using BLAST Software (http://www.ncbi.nlm.nih.gov/BLAST). All HCV genotypes were defined with a query coverage of ≥98%, analysing at least 170 nucleotides of the target sequence.

### Mitogen *in vitro* Stimulation of PBMCs

To investigate the replication and potential transmission risk of OCI, we performed a 7-day mitogen *in vitro* stimulation of PBMCs collected from OCI subjects both at recruitment and during follow-up, and assessed and quantified the release of HCV virions in the supernatant. PBMCs from one HCV-positive subject with an HCV viral load of 3.70×10^5^ IU/ml, and from two HCV-negative subjects were analogously stimulated to be used as positive and negative controls, respectively. Fresh or stored alive PBMCs were cultured *in vitro* in RPMI supplemented with 10% FCS, under mitogen stimulation with Interleukin-2 (20 U/mL) and phytohaemoagglutinin (5 µg/mL), according to previously described methods [2]. About 10×10^6^ fresh or 5×10^6^ thawed from storage PBMCs were incubated with mitogens at 37°C with 5% CO_2_ for 7 days. Afterwards, cells were harvested and counted, and supernatant after ultracentrifugation was investigated for HCV-RNA detection by reverse-transcription nested-PCR.

### Statistical Analysis

We analysed three separate groups of subjects: the ILDF group included all subjects negative for serological markers of both HBV and HCV infection (HBsAg, anti-HBc, anti-HBs, and anti-HCV; HBV-DNA and HCV-RNA), the HCV-positive control group included subjects with confirmed HCV infection (anti-HCV-positive), the HBV-positive group included anti-HBc-positive subjects and the active HBV infection subgroup included subjects with markers of HBV infection in serum (HBsAg or HBV-DNA) (Figure 1, Table 1).
